# Visualization and Analysis of Multidimensional Cardiovascular Magnetic Resonance Imaging: Challenges and Opportunities

**DOI:** 10.3389/fcvm.2022.919810

**Published:** 2022-07-04

**Authors:** Leon Axel, Timothy S. Phan, Dimitris N. Metaxas

**Affiliations:** ^1^Department of Radiology, New York University Grossman School of Medicine, New York, NY, United States; ^2^Department of Computer Science, Rutgers University, Piscataway, NJ, United States

**Keywords:** cardiovascular, magnetic resonance imaging, MRI, multidimensional, visualization, analysis

## Abstract

Recent advances in magnetic resonance imaging are enabling the efficient creation of high-dimensional, multiparametric images, containing a wealth of potential information about the structure and function of many organs, including the cardiovascular system. However, the sizes of these rich data sets are so large that they are outstripping our ability to adequately visualize and analyze them, thus limiting their clinical impact. While there are some intrinsic limitations of human perception and of conventional display devices which hamper our ability to effectively use these data, newer computational methods for handling the data may aid our ability to extract and visualize the salient components of these high-dimensional data sets.

## Introduction

While cardiovascular magnetic resonance imaging (CMR) has become a valuable clinical tool, conventional CMR still has many limitations. Recent ongoing advances in the development of CMR imaging methods are now making possible the acquisition and reconstruction of much more comprehensive sets of imaging data on the cardiovascular system, including the creation of large-scale multidimensional and multiparametric images. However, limitations of the human perceptual system and conventional display devices make the visualization and analysis of these large and complex data sets challenging. We will briefly summarize some of the background related to the creation and potential applications of these new CMR data sets, and we will discuss some of the associated challenges involved in handling them, as well as some possible paths forward to meet these challenges.

## Conventional Cardiovascular MRI

Magnetic resonance imaging (MRI) uses the physical phenomena of nuclear magnetic resonance to create images that reflect multiple aspects of the state of the body, including the following principles: (1) Certain nuclei, including hydrogen (one of the principal constituents of the body), can become magnetized in a strong magnetic polarizing field, producing a collective bulk nuclear magnetization. (2) The orientation of the nuclear magnetization relative to the polarizing field can be changed (“excited”) by applying an oscillating magnetic field at a specific resonance frequency (proportional to the strength of the polarizing field); if the nuclear magnetization is left at an orientation inclined to the field direction, it will produce a weak, but detectable, signal at the resonance frequency. (3) Position information can be encoded in the signal, through the use of supplementary magnetic fields which vary with position in a controlled way (“gradients”), with associated changes in the local resonance frequency (1); the resulting signals are equivalent to samples of the Fourier transform of an image. A suitable set of such encoded signals can be used to reconstruct an image (2D or 3D) of the spatial distribution of the signal sources, through an inverse Fourier transform. Gradients can also be used during excitation to select a desired plane to image. Serial images over time can display motion. (4) Tissue state-dependent “relaxation times” can affect the strength of the signal; additional excitations can be used to change the local image contrast, through relaxation time-dependent effects on the signal, which can help to reveal the presence of abnormalities in the image, or to calculate the regional relaxation times or other parameters (“parameter mapping”), typically by measuring the difference in image intensity produced by the additional excitations. Such parameter mapping can potentially be used for more quantitative characterization of disease states. Contrast agents alter the image appearance by altering the local relaxation times; early and late contrast enhancement patterns provide information on perfusion and associated tissue abnormalities, which can also potentially be quantified. (5) Additional gradients can be used to sensitize the signal to motion effects, including velocity and diffusion, enabling flexible measurements of blood flow and providing an additional potential means for tissue characterization. (6) Nuclei at different positions within a molecule may have slightly different resonance frequencies, potentially providing some chemical information in the signal, e.g., distinguishing fat from water.

In applying MRI to the cardiovascular system, we have to deal with the effects of motion on the images, related to both the heart beat and breathing. If the heart beats are sufficiently similar, we can pool data from multiple heart beats, to create “cine” images at multiple relative times spanning an averaged cardiac cycle. If data acquisition times are short enough, images can be created during suspended respiration, to eliminate respiratory motion effects. In conventional clinical cardiovascular MRI, sets of relaxation time-weighted and cine images, acquired in multiple planes, are used to evaluate the local and global structure, tissue properties, and function, primarily through qualitative assessment of 2D images.

While they are clinically very useful, there are still significant limitations of conventional CMR methods. Due to breath hold limits, most imaging is 2D and acquired with separate breath-holds, leading to long imaging sessions needed to cover the heart and vessels, and potential position inconsistency between images, due to inconsistent breath-holds. Internal inconsistency of the acquired data, e.g., when patients cannot suspend respiration or have arrhythmia, can lead to image degradation. Conventional parameter mapping is time-consuming and vulnerable to motion and other artifacts, limiting its clinical use.

## Newer CMR Methods

**Figure 1:**
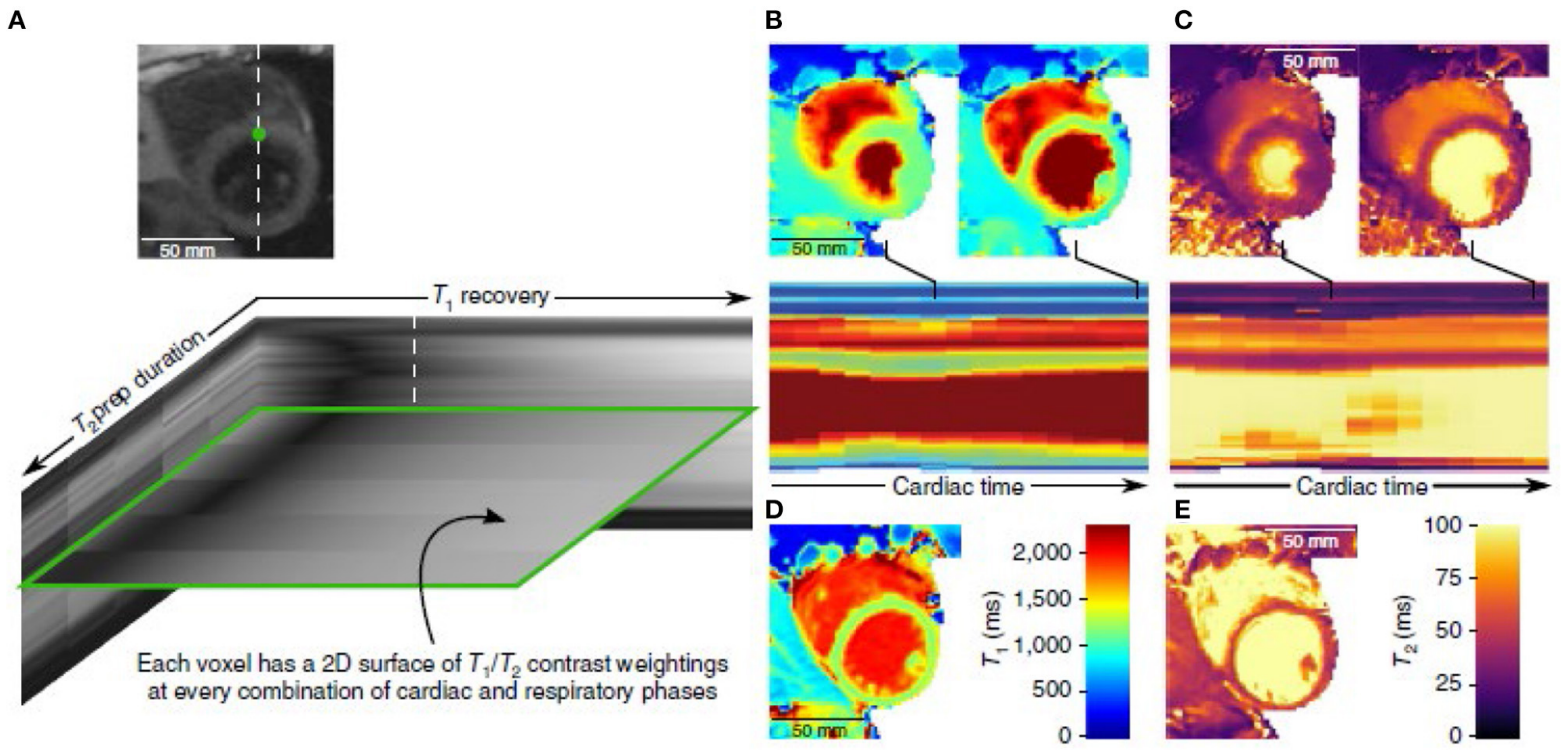
Example of the use of multitasking imaging, with use of simultaneous T1- and T2-weighting **(A)**, to generate motion-resolved multidimensional images with parameter mapping **(B–E)** [(4) (by permission)].

Various approaches have enabled the use of undersampling of the imaging data, taking advantage of the underlying correlations between pixels in medical images (“compressed sensing”), thus shortening imaging times. In addition to accelerating conventional imaging, these new methods make it possible to acquire larger image data sets in a reasonable time. In the context of CMR, considering cardiac and respiratory cycle phases as effectively being additional dimensions enables reconstruction of respiratory- and cardiac-synchronized 2D and 3D image data sets from free-breathing continuous data acquisitions (2, 3). Previously, parameter mapping required acquiring multiple images with different degrees of “steady” sensitivity to the desired tissue property, and then combining them to calculate the value of the property; however, this is a time-consuming process, and is subject to errors related to any position inconsistency between the images. Newer mapping imaging methods have taken a more efficient dynamic approach, directly incorporating the “unsteady” response of the signal to transient perturbations, such as additional excitations or contrast injections, into the data acquisition process. The resulting mixed effects on the signals can then be separated during the image reconstruction process, using mathematical tools such as “low rank” decomposition of the resulting image data set. This “multitasking” approach enables adding parameter mapping and perfusion imaging to a combined image reconstruction. Such combined multidimensional data acquisition and reconstruction imaging methods can thus enable direct joint creation of images of regional tissue properties, together with the motion, without the conventional need to separately reconstruct the properties from sets of sequential data acquisitions (which are vulnerable to problems from inconsistent tissue positions) (e.g., Figure 1). An alternative approach to multidimensional and multiparameter imaging is to use the evolving response of the signal to continuously varying excitation pulses and gradients (“fingerprinting”). However, all these approaches have previously required time-consuming associated iterative image reconstruction methods; machine learning (ML) methods are a promising way to speed up this up (4–10), and ML-based methods are being rapidly developed for this and many other image-related applications. Artificial intelligence (AI)-based methods can be used to help suppress artifacts that may arise from more conventional image reconstruction methods, e.g., due to motion or data undersampling. However, AI-based methods for image handling programs may be subject to instabilities. These and related methods are described in more detail and illustrated in the accompanying articles in this issue, and will not be further discussed here. We can also potentially acquire multinuclear imaging data, for an additional set of “dimensions” to display and analyze.

One thing that these methods all have in common is the ability to create very large and high-dimensional data sets; it is very challenging to visualize and analyze the wealth of data that they can potentially contain. The same underlying correlations between the images across the multiple images that make it possible to reconstruct them from undersampled data also make it possible to represent them in a correspondingly compressed form. This can make for a more compact way to store the data, and the images can then be selectively re-expanded, as needed, for displays, making for more efficient data handling. Beyond the immediate challenge of how to adequately and interactively survey the full extent of such large data sets, there are also challenging quantification issues related to associated tasks in handling the data, such as segmentation of different structures, characterization of time evolution across multiple dimensions, and identification/classification of abnormalities revealed by regional alterations in different parameters. Segmentation of cardiac structures is already challenging for conventional CMR functional analysis; segmenting cardiac structures across multiple dimensions compounds that challenge. However, being able to quantitatively assess the interactions between cardiac and respiratory cycles potentially offers new ways to characterize cardiovascular function (11, 12). One promising approach to the challenges of multidimensional image handling is the napari project, which aims to develop an open-source set of high-performing multidimensional image display and analysis tools.

## Human Perception Limitations

We live in a 3D dynamic world; our visual interactions with it are mediated through images projected onto effectively flat retinas. We infer relationships between 2D object features that we see in an image and the corresponding underlying 3D object surface *via* visual cues, such as shading and occlusions, and from stereo disparities of details between left- and right-eye views. However, the structures captured in our 3D (or higher dimensional) MR images may not have well-defined implicit discrete surfaces to render; this is a frequent problem with conventional 3D medical imaging methods, when trying to display such structures as 3D objects. We can explore a 3D data set by interactively scrolling through consecutive 2D sections through it, or by displaying an array of multiple such images, but it is difficult to directly compare different regions with such displays. One common way to try to effectively compress 3D data of the heart into a 2D display is to create target-like “bull's eye” plots, e.g., with concentric rings representing different short-axis locations in the ventricle walls from apex to base and a color scale linked to some mapped quantity; however, this is associated with decreased data sampling density and geometric distortion of the displayed structures.

Our eyes have perceptual limitations, including a limited dynamic range and a limited ability to discriminate similar intensities. Vision is also affected by simultaneous contrast; that is, the subjective appearance of a region can be significantly altered by changes in the brightness or color of surrounding regions. We are not good at visually judging absolute brightness. We also cannot readily attend to multiple properties and different locations at once, and thus have difficulty comparing corresponding regions between different kinds of separately displayed images. These limitations are further exacerbated when we need to compare and register motion patterns in separately displayed images of different locations. These perceptual limitations are already a problem with conventional intensity-based imaging, and they become more acute when trying to incorporate additional parameters into the images.

## Display Options and Limitations

Conventional computer displays use flat screens, which is already a significant limitation for viewing 3D data. The usual approach for interactively viewing 3D data is to use “multiplanar reformatting” (MPR) to create a virtual view of an interactively selected 2D plane from within the 3D volume; however, while MPR is very useful, it can still be difficult to build up a reliable understanding of the underlying 3D structure relationships in the imaged volume from viewing such sampled 2D slices. An alternative display approach is to use a “volume rendering technique” (VRT) approach to display a shaded rendering of implicit “surfaces” within the 3D volume; newer “cinematic” approaches to generating the shaded surface displays can help make the spatial relationships of the displayed structures clearer. However, the structures of interest in the imaged volume may not have sufficiently sharp boundaries in the intensity data to enable their use for such rendering. When extending these approaches to time-varying (e.g., real or “physiologic” time) or other multi-dimensional data, we can use interactive scrolling through or animation of a corresponding set of such displays over time or other dimensions. For images that evolve over time, e.g., images of dynamic contrast enhancement, it may be more useful to display functional images that effectively summarize the time evolution of the signal, e.g., by calculating the temporal moments or perfusion-related variables, rather than displaying the multiple underlying serial images, themselves. However, when additional dimensions are introduced into the data, we must choose which dimension to animate, as only one dimension can be mapped into the animation at a time, and we may still have difficulty exploring interactions between the different dimensions. The task of interactively exploring all potentially relevant areas of a large multidimensional data set can be daunting (like “looking for a needle in a haystack”).

For display of single imaged parameters, or of scalar variables calculated from the image sets, we can use a simple color overlay with varying opacity onto the corresponding underlying intensity image data; we can interactively adjust the associated color and opacity lookup tables to qualitatively bring out structures of interest in the display. When dealing with multiparametric image data, we must choose a given property (or combination of properties) to map (using an associated color lookup table) for a given display. However, things get more difficult when we want to examine the spatial distribution of more than one scalar property at a time. Although we can try to use some sort of hue/saturation/value mapping to display multiple properties at once, these effective display “dimensions” are not very “orthogonal” to each other, and the eye's response to them is not very linear. An alternative approach to jointly evaluate images of multiple parameters is to enable interactive exploration of different combinations of the parameters. For example, we can create synthetic images reflecting the expected appearance of images that would have been acquired with different relative amounts of parameter weighting; this may be of more practical utility than simple images of the parameter values themselves. One way to approach this would be to map up to three different coregistered parameter values to be displayed in separate red, green or blue overlaid colors, with the net perceived color reflecting the relative contributions from each parameter. Alternatively, a principal component analysis approach could be used to look for ways to combine multiple parameters that would best distinguish between different particular tissue states. As an example, this approach could potentially be used to help distinguish myocardial regions of bright appearance on T1-weighted imaging that are due to late gadolinium enhancement, rather than to fat or residual contrast enhancement of the adjacent blood, which would have different chemical shift or T2 values than enhanced myocardium. Qualitative assessment of the spatial variation of such multiparametric-based displays may be more clinically useful for identifying and classifying regional abnormalities than simple local measurement of specific numerical values of parameters.

Motion and other temporally varying properties are often not readily summarized as simple scalars, although approaches such as calculation of associated temporal moments can be useful. Higher-dimensional imaged properties, such as vectors (e.g., velocity) and tensors (e.g., deformation or diffusion), can be discretely represented with arrows or glyphs at sampled locations; however, it is difficult to appreciate their 3D orientation and scales from a flat image, although “motion parallax” effects seen while interactively changing the view orientation can help. The velocity fields can also be used to generate corresponding streamlines or virtual particle traces (13). To aid the 3D representation of such higher order variables, we can let arrows or glyphs closer to the viewer progressively occlude those behind them, or create an orientation-dependent appearance for their representation, but this is inherently a difficult task. It is already challenging to work with conventionally acquired 4D (3D plus time) flow/motion data for display and analysis; adding additional effective dimensions will only compound this difficulty. While virtual reality (VR) display tools with stereoscopic capabilities have been used to augment the conventional visualization of 4D flow data, some users have found them to induce nausea, and the associated available image display resolution is still relatively limited, indicating that the virtual display technology may still need more development before it is ready for adoption for clinical use.

## Potential Ways Forward

Another area of similarly high information content imaging is multispectral or hyperspectral imaging, e.g., used for remote sensing of the environment or astronomy. Thus, we could potentially adapt methods used with hyperspectral imaging for handling multiparametric MR images; for example, we can seek to use linear principal component analysis or, for improved results due to the inherent non-linear nature of the problem, use machine learning (ML) approaches to combine data with different parameters for particular tissue characterization purposes.

Although they are still undergoing technical development, as described above, we can potentially adapt VR displays to enable better understanding of 3D spatial relationships within the multidimensional CMR data. However, this still effectively only incorporates one additional dimension in the display.

We will likely need to develop and apply ML-based image analysis methods, in order to improve the automated discovery of salient regions/manifolds of the non-linear higher-dimensional “spaces” of multidimensional/multiparametric CMR, which can then be used for guided exploration of the data. As we have not previously had direct access to such kinds of high-dimensional data, developing the initial annotated data sets needed for the training of such methods will still be challenging. Thus, we will need to initially acquire and analyze such multidimensional data on a sufficient number of normal subjects to be able to establish regional normal ranges for such data, and we will need to be able to register individual patient image data sets to such normal values data. After the use of the initial supervised machine learning methods, we can use unsupervised machine learning methods for cardiac analytics which do not require data annotations, such as those recently developed by our group (14, 15); this is now an area of active research by many groups.

An advantage of these newer multidimensional CMR methods is that they can provide the data on different parameters in mutually spatially-registered ways. However, different components of some data sets are likely to still have different levels of signal-to-noise ratio (SNR) or different spatial resolutions, e.g., with multinuclear imaging. Thus, we will need to find effective ways to combine data derived from the higher SNR and resolution components of the imaging, e.g., for definition of regions of interest for quantitative analysis, with the other lower “quality” image components, for better integrated analysis of the data.

As with conventional parameter mapping, quality assurance and standardization of the data resulting from multidimensional imaging will be needed before the results can be trusted enough to be relied upon for clinical decision making. Imaging of phantoms containing material with calibrated parameter values can provide a minimum standard for such evaluation, but may not adequately test for effects on the data of *in vivo* imaging, such as due to motion. The validation of data related to motion-related analysis of multidimensional images is also necessary but challenging. Imaging of physical or numerical phantoms with known motion properties, while useful, may again not adequately assess the potential effects of *in vivo* imaging on the derived numbers. The wide range of potential approaches to the display of multidimensional data is a strength, but it will make standardization of the displays more difficult.

An “ideal” viewing/analysis user interface for the display of multidimensional/multiparametric CMR image data would provide a set of fast and flexible interactive tools for exploring the data set. These could include: (1) freely “cutting into” the different parametric components of the data with MPR, (2) the ability to freely move along or animate different time-related dimensions, (3) the ability to flexibly synthesize new combined displays from the component parameter images, (4) interactive creation of MPR and VRT images from any of these kinds of displays, and (5) options to use VR tools for an enhanced understanding of 3D spatial relationships of the displayed structures.

## Discussion

These new multidimensional/multiparametric CMR methods have great clinical potential, through their ability to efficiently create spatially registered images of multiple regional structure, function, and tissue properties. However, due to their large size and complexity, they also present multiple challenges related to their effective display and analysis. Limits posed by human perception, both in viewing displays and in integrating the multidimensional data, impede our ability to fully grasp the high levels of information that can potentially be contained in these new data sets. Conventional display technology methods and visualization techniques also have many limitations that can restrict our ability to interact with these data. ML-based methods are undergoing rapid development in many areas; in addition to aiding the reconstruction of these large data sets, we may be able to incorporate some aspects of their analysis directly into the reconstruction process, such as automated segmentation of cardiovascular structures and recovery of functional variables, as well as identification and classification of regional abnormalities.

There is a great potential for gaining additional clinical value from the multidimensional/multiparametric data produced with these new imaging methods, once we develop appropriate methods to handle the associated challenges of visualizing and analyzing them.

## Data Availability Statement

The original contributions presented in the study are included in the article/supplementary material, further inquiries can be directed to the corresponding author.

## Author Contributions

All authors have contributed to this manuscript, and they have reviewed and approved the final version of the manuscript.

## Funding

This work was partially funded by NIH grants 1R21EB029168 and 5R01HL127661.

## Conflict of Interest

The authors declare that the research was conducted in the absence of any commercial or financial relationships that could be construed as a potential conflict of interest.

## Publisher's Note

All claims expressed in this article are solely those of the authors and do not necessarily represent those of their affiliated organizations, or those of the publisher, the editors and the reviewers. Any product that may be evaluated in this article, or claim that may be made by its manufacturer, is not guaranteed or endorsed by the publisher.
